# Factors influencing delays and overtime during surgery: a descriptive analytics for high volume arthroplasty procedures

**DOI:** 10.3389/fsurg.2023.1242287

**Published:** 2024-01-04

**Authors:** Farid Al Zoubi, Paul E. Beaulé, Pascal Fallavollita

**Affiliations:** ^1^ School of Electrical Engineering and Computer Science, University of Ottawa, Ottawa, ON, Canada; ^2^ Division of Orthopedic Surgery, Ottawa Hospital Research Institute, Ottawa, ON, Canada; ^3^ Interdisciplinary School of Health Sciences, University of Ottawa, Ottawa, ON, Canada

**Keywords:** descriptive analytics, health care efficiency, high volume surgery, operating rooms throughput, overtime hours

## Abstract

The aim of this article is to analyze factors influencing delays and overtime during surgery. We utilized descriptive analytics and divided the factors into three levels. In level one, we analyzed each surgical metrics individually and how it may influence the Surgical Success Rate (SSR) of each operating day. In level two, we compared up to three metrics at once, and in level three, we analyzed four metrics to identify more complex patterns in data including correlations. Within each level, factors were categorized as patient, surgical team, and time specific. Retrospective data on 788 high volume arthroplasty procedures was compiled and analyzed from the 4-joint arthroplasty operating room at our institution. Results demonstrated that surgical team performance had the highest impact on SSR whereas patient metrics had the least influence on SSR. Additionally, beginning the surgical day on time has a prominent effect on the SSR. Finally, the experience of the surgeon had almost no impact on the SSR. In conclusion, we gathered a list of insights that can help influence the re-allocation of resources in daily clinical practice to offset inefficiencies in arthroplasty surgeries.

## 1 Introduction

The term arthroplasty is the amalgamation of Arthro (Greek), meaning joint, and Plasty, which means to mold, graft, or reform. Hence, arthroplasty is the science of molding or reforming a joint, usually to reclaim its full function or relieve joint pain (1). Human joints become painful and stiff (with age) from regular wear and tear alone, but certain degenerative diseases can exacerbate the condition. Arthroplasty surgeries are the ultimate corrective measure to rectify these conditions. The procedure may include retaining the healthy parts of the joints and augmenting them with implants (i.e., resurfacing, partial replacement procedures) or completely replacing the joint (both ends) with implants.

To address issues with hospital efficiency, various initiatives to increase throughput, such as high-efficiency operating rooms (ORs) and parallel processing with anesthesia block rooms, have been suggested (2). At our hospital, we instituted increased through put rooms going from two-three to dedicated four primary joint rooms with dedicated arthroplasty surgeons in. The 4-joint OR was designed specifically to handle these procedures, and everything, from its layout to equipment, has been arranged with arthroplasty surgeries in mind. The design allows surgeons and staff to save time on procedures and complete more surgeries in a day than a general OR handling different types of surgical patients would allow for.

In the area of arthroplasty, there exists few works that identify factors which influence surgical outcomes. Authors in (2) determined that patient length of stay is multifactorial and can be reduced by regular review of the care pathway to effect incremental changes that have been identified as having an impact on reducing stay. In (3), authors identified specific factors that ensure positive patient outcomes following knee surgery, both non-surgical (i.e., gender, age, body mass index, etc.) and surgical factors (i.e., anesthesia, postoperative complications, and rehabilitation). Lastly, authors in (4) concluded that patients' perception of pain control was significantly positively correlated with the perception of their orthopedist, nurse, and overall hospital satisfaction.

Different to the state-of-the-art, the objectives of our study are to identify the factors which influence Surgical Success Rate, or SSR, which is the ratio of successful surgery days over total surgery days. To our knowledge, this is the first attempt at identifying factors which contribute to surgical delays/overtime in the application area of orthopedics. The definition of successful surgery days (in this context) is a day in which all four joint surgeries scheduled are completed within the dedicated eight hours (between 7:30 am and 3:30 pm). An unsuccessful day has two negative consequences:

1. Overtime, which costs our institution $570,000 a year (5). The dollar amount was calculated by multiplying the number of additional minutes an OR was engaged for (during unsuccessful days = 10,179 minutes) by $56, which is the per-minute collective cost of an OR and the staff using it.
2. Postponing the fourth patient of the day to a future date. This results in backlog, low patient satisfaction rates, and underutilization of hospital resources with the third case often ending at 14:30–15:00, let alone the unused time for days that team manages to complete their fourth case before 15:30. This unused time is calculated to be an average of 36 min per successful day in our institution.

The following sections of this work are divided into three levels based on the number of surgical variables being analyzed simultaneously, for which level one is the simplest format of analysis while level three is the most complex analysis.

## 2 Descriptive analytics

Descriptive Analytics (DA) is the science of analyzing available data to determine patterns and trends. It focuses on “what happened?”, not how, why, or if it might happen again. It's relatively easy to understand, which makes it useful and accessible to a wider audience. It can offer a wealth of useful insights and help with decision-making (6).

The primary advantage of descriptive analytics is that it allows you to view how certain variables, relationships, and trends change over time. This, along with its simplicity, makes it quite useful to evaluate and communicate performance. Anything that can be quantified via metrics, changes over time, and has discernable patterns, is in the realm of descriptive analytics. Another advantage it offers is easy-to-comprehend visualization of complex numerical data, which makes it more palatable for people from different departments and disciplines. More eyes on data can help with more insights and unique perspectives, which aid in decision-making (7).

Descriptive analytics has multiple use cases in healthcare (8). It's used for trend analysis, such as identifying which age group and BMI category has the most joint replacement surgeries (9). It also assists in planning, such as stocking up on flu vaccine before certain months of the year, based on past trends (10). Descriptive Analytics can also lead experts to the right causes behind certain trends just by helping them realize what to look for.

Multiple types of descriptive analysis techniques and approaches are associated with both individuals and clinical units (7), including case reports (11), cross-sectional studies (12), and surveillance (13). For our study, we are following the passive surveillance approach for descriptive analytics, i.e., systematic collection of data pre-divided into formal categories and spread out over an adequate period of time (i.e., enough for cyclical patterns to emerge).

The retrospective data we have compiled and analyzed for this study comes from the 4-joint operating room for arthroplasty procedures at our institution. The critical characteristics of the data are as follows:

• Time Period: 2012–2020
• Nature of Procedures: Non-complex cases, Unilateral hip and/or knee replacement surgeries only.
• Nature of Data: Numerical (mostly time stamps and time durations) and Categorical
• Data Collection Source: Surgical Information Management System (SIMS) for the majority of the metrics, while some other were collected manually from daily notes.
• Data Dimensions: 26 Columns and 788 rows. See Supplementary Table S1 for all metrics.
• Treatment of Data: Data was cleaned for missing information and incorrect values, both of which represented less than 1% of the observed dataset. Rare cases, regardless of their dissent with median values, were kept in the dataset.
• Surgical Success Rate (SSR): was calculated at 39%.
• Demographics and Other Quantifiable:
  ▪ Number of days and surgeries: 197 days (788 surgeries)
  ▪ Number of surgeons: 6
  ▪ Number of Nurses: 73
  ▪ Number of Anesthesiologists: 81
  ▪ Gender-wide Distribution of Sample Patients: 385 M, 403 F
  ▪ The average age of patients: 63.2 ± 11.9
  ▪ Average BMI: 30 ± 5.7

Time intervals used are a modified version of those defined by the Association of Anesthesia Clinical Directors (AACD): anesthesia preparation time (APT); patient in-room to anesthesia ready, surgical preparation time (SPT); anesthesia ready to procedure start, procedure; procedure start to procedure finish, anesthesia finish time (AFT); procedure finish to patient out of room, and turnover time; and first patient exits to subsequent patient in room. APT immediately follows turnover. Figure Below illustrates the surgical intervals along with their spans.

## 3 Level-1: SSR vs. individual metrics

At Level-1, we are analyzing each metric individually and how it may influence the SSR. Analyzing SSR from the perspective of each metric can help us identify outliers, irrelevant factors, and trends that may otherwise get buried under the data. Another benefit of focusing on individual metrics is the ability to weigh each metric for its influence on SSR or, at least, identify metrics with the most significant and least significant impact on SSR, Supplementary Table S2. Less resource-intensive and high-impact metrics can help us develop intervention strategies that may directly reflect in a high SSR. Conversely, more resource-intensive, high-impact metrics can become the elements of a more comprehensive, long-term strategy to improve the success rate.

We have divided the metrics of Level 1 of analysis into three different categories:

1. Staff and facility-specific metrics: calculating the impact of controlled and managed resources (i.e., primarily human resources) on SSR.
2. Patient metrics: calculating the impact of these metrics on the SSR offers great insights regarding patient management and scheduling, especially with the data/insights we have access to from the first category.
3. Time-related metrics: identifying which aspects of the surgeries have the most significant impact on the success rate and their timely completions. This helps with the development of multi-faceted optimization strategies covering both individuals and processes to improve SSR.

### 3.1 SSR vs. staff and facility

#### 3.1.1 SSR vs. campus

Our institution has two campuses: Civic and General. The bulk of the surgeries happen at the General; with a 7:1 ratio of surgeries. There is only a 4% difference in the SSR, as 36% of the Civic Campus's surgeries are successfully compared to 40% at the General Campus, average SSR is 39%. The difference is not nearly as significant as the difference in the number of surgeries, and no other metric supports the assumption that a higher number of surgeries resulted in a higher SSR (see Supplementary Figure S2).

#### 3.1.2 SSR vs. surgeon

The SSR varies greatly from one surgeon to another. The lowest extreme is 13.3% SSR (if we neglect the seventh surgeon with a 0% SSR), and the highest extreme is 62.3% (see Supplementary Figure S3). One curious observation from this comparison is that there is no discernable connection between the number of surgeries and SSR, i.e., SSR is not tied to the experience gained from performing more surgeries. Surgeons S3 and S4 have a minimal difference in the number of surgeries they conducted, but the SSR difference is significant 41.1% vs. 62.3%. This is further endorsed if we compare the three closest SSRs for surgeons S4, S5, and S6. S6 completed roughly 4.6 times, and S4 completed 6 times more surgeries than S5, but the SSR difference is minimal. This is also not a true reflection of a surgeon's capabilities, at least not without taking other factors like team and surgery type into account. However, it may help identify the best performer and worst performers if their difference from the mean is significant enough.

#### 3.1.3 SSR vs. anesthesiologist

Like the result above, the SSR is not correlated to the number of surgeries an anesthesiologist has been a part of. In fact, the opposite is more plausible, i.e., the higher the number of surgeries, the lower their SSRs might be (Supplementary Figure S4). This is supported by the fact that there is just one anesthesiologist with a 100% SSR that completed more than ten surgeries and Supplementary Figure S5: Circulating nurse's experience does not influence SSR.at least fifteen anesthesiologists that completed less than ten surgeries. The anesthesiologists with high SSRs can be considered a controlled factor for future surgeries to influence the probability of a surgery succeeding on time. However, to determine the potency of this controlled factor, it's imperative to take the influence of an anesthesiologist on a surgery completed on time into account.

#### 3.1.4 SSR vs. circulating nurse RN1 and RN2

The number of surgeries a registered nurse (RN) attends to does not influence the SSR, and the fewer surgeries an RN has attended to, the higher their chances of achieving a respectable SSR. However, it's difficult to identify discernable trends because of the statistical weaknesses of this dataset or, more accurately, its distribution (Supplementary Figure S5). The top 3 RN2s and top 4 RN1s have completed more surgeries than the rest combined. The uneven distribution of sample data makes it impossible to identify the connection between a RN and SSR.

### 3.2 SSR vs. patient metrics

A patient's physical condition, the type of surgery they need, their age, and gender can have a significant impact on the successful completion of surgery on time.

#### 3.2.1 SSR vs. sex

The SSR for Male patients is slightly higher than for male patients (Supplementary Figure S6). It's consistent with the finding of another study that investigated the operative times of surgeries for male vs. female arthroplasty patients. A study has demonstrated that men are at higher risk of developing prosthetic joint infections following joint arthroplasty, thus surgeons have to take extra precautions during surgeries (14). However, we believe that the real practical reason is that men are more muscular which makes surgery more difficult. This insight can be used for patient scheduling to improve the overall SSR. Scheduling two males and two females per day, or scheduling three or four females in one day when there are surgeries with anticipated complications, can be strategically helpful to make up for time delays and enhance the chances of completing four surgeries in the allotted time.

#### 3.2.2 SSR vs. age

The bulk of the age-wise SSR trend hovers between 32% and 46%, with one outlier being the age group between 17 and 26. The youngest group also has the lowest number of surgeries, and it's consistent with typical age-oriented surgical recovery and success trends. But apart from that, there is no discernable trend. There is an 8% difference in the SSR for people between the ages of 57 and 66 and patients between 27 and 36 years of age, with older patients having a higher SSR (Supplementary Figure S7).

#### 3.2.3 SSR vs. BMI

The BMI correlation with SSR offers pattern abnormalities similar to age (Supplementary Figure S8). It's highest for patients in the Class 3 obesity BMI. This is inconsistent with the observation for both elective surgeries like Total Knee Replacement (TKA) and Total Hip Arthroplasty (THA).

#### 3.2.4 SSR vs. ASA

The primary concern with identifying patterns when comparing SSR with the American Society of Anesthesiologists (ASA) physical status classification system classes is the data distribution. The sample sizes of Class I and IV are lower compared to Class II and III. If we average out Class II and III (about 367 cases), Class I is 10.3%, and Class IV is 3.4% of that sample size. Between two reasonably comparable classes (II and III), the pattern is as expected—higher for a safer ASA class and lower for a riskier class (see Supplementary Figure S9).

### 3.3 SSR vs. time stamps

#### 3.3.1 SSR vs. months

An interesting pattern was observed when we analyzed SSRs for different months of the year. Apart from two exceptions (July and August), the remaining ten months can be divided into sets of two. Five of them are above 50%, and five are between 30% and 40%. May, the month with the highest SSR, is a true outlier, and August, the month with the lowest SSR, is the culmination of a four-month-long downward trend. The variation in the number of surgeries for each month is also a pattern worth considering, as it may be tied to factors like staff availability and fatigue (15). However, it doesn't impact the surgical success rate as both the highest and lowest SSR months had only a difference of about ten surgeries, which is less than 15% of total surgeries for either month (Supplementary Figure S10).

#### 3.3.2 SSR vs. days

The SSR for days shows that the best days for surgery are one day after the weekend ends and one day before the weekend begins, i.e., Tuesday and Thursday. Also, for days, the pattern of more surgeries resulting in a higher SSR holds apart from one outlier (Monday). This could be construed that it's tough to get to work on Monday, and on Fridays' the majority of people are looking forward to the weekend which may influence their focus on surgery (Supplementary Figure S11). One study shows that employees are less supportive on those days, i.e., Monday and Friday (16).

#### 3.3.3 SSR vs. time in room and anesthesia ready time

The correlation between Time in Room and Anesthesia Ready Time is evident from the SSR pattern for both variables, and it's tied to the starting time of the surgery (Supplementary Figure S12). The SSR is higher for surgeries in which the patient was in the room and anesthetized closer to the scheduled time/allotted time slot. The farther away they were from that time window, the lower their SSR became. For example, if by 8:20 AM (Cut-off Time) the first patient (P1) was not in the OR already, there is no way the fourth surgery can be completed without having to pay overtime. Another example is that if the third patient (P3) did not have his anesthesia ready by 1:00 PM, there will be a very slim chance (less than 20%) the fourth surgery would be completed on time, i.e., before 3:30 PM. As an observation, the nurses arrive to work at 7:30am and there is no real accountability for that first 25–29 min in terms of productivity as long as the patient is in the room before 8:00 am and as one would expect this often spill into after 8:00 am.

#### 3.3.4 SSR vs. case start and case finish time

A similar pattern was observed when we compared SSRs against Case Start and Finish times. The procedures that started and ended in the allotted time slots had a much higher SSR rate. The four waves in Supplementary Figure S13 represent four-time slots for four arthroplasty surgeries in a given day, along with the cut-off time for each wave where the patient should be no later to consider it as a successful day.

#### 3.3.5 SSR vs. time out of room and anesthesia stop time

The pattern is the same for SSRs when compared against Time Out of Room and Anesthesia Stop Time—four waves endorsing the observation that surgeries that start on time and end on time resulted in higher SSR days (see Supplementary Figure S14).

#### 3.3.6 SSR vs. anesthesia start time and turnover

The anesthesia start time doesn't conform to the same pattern, at least not with the same degree of correlation, as other time metrics when compared to SSR. A downward pattern is observed between SSR and turnover rates (i.e., the time to prepare the room for the next surgery), but it includes a hard spike and a relatively hard slump. On the other hand, turnover is measured in minutes, thus Supplementary Figure S17 represents duration rather than time stamps. Turnover time should take between 12.9 and 17.9 min to achieve the highest SSR, considering the 42.9–47.9 window as an outlier since there are not enough samples to support this high SSR.

## 4 Level-2: comparing timestamps vs. patient metrics

As we analyze a level deeper, we are comparing two to three variables at once. It's mostly a three-dimensional analysis compared to Level-1, where we compared one to two variables/metrics side by side to determine a pattern. It's also different from the Level-1 time-metric comparisons because it compares averages to patient metrics instead of timestamps.

### 4.1 Time vs. age

The average procedure time and the average case total time follow an almost parallel pattern since procedure time makes up the bulk of the case total time. Interestingly, the turnover rate follows a similar pattern. The Anesthesia Preparation time (APT) average is the most obvious outlier, as the average consistently goes up until the second last age group and then drops off. If we observe the averages excluding the two extremes, it's clear that Anesthesia preparation and in-room time go up with age. For patients above 45 years old, the chances of spending more time in the OR become higher as they reach 76 years old and steadily lower after 76 years old (Supplementary Figure S16).

### 4.2 Time vs. BMI

The averages for APT in Room, Surgery Finish time (SFT), and turnover have gone up with the BMI. In contrast, averages for Surgery Preparation Time (SPT) and procedure went down as BMI increased. The Case Total average time is most significantly influenced by the procedure average time and APT average, which rises sharply with BMI but drops off for the riskiest BMI class (Supplementary Figure S17). It also shows the APT's influence on Case Total, which followed the APT's trajectory instead of the procedure averages, between the BMI of 27.1 and 47.1.

### 4.3 Time vs. sex

On average, surgeries for male patients takes 5 min longer than surgeries for female patients. The SPT and SFT averages for males are also slightly higher (one minute on average), which pushes the total time difference (Case Total) to six minutes (Supplementary Figure S18). This is one rationale behind the higher SSR for female patients as shown in Section 3.2.2.

### 4.4 Time vs. ASA

Anesthesia-related averages are following the naturally expected pattern, i.e., moving up for higher/riskier ASA classifications, though there is virtually no difference between Class I and II. The pattern for the average Case Total and Procedure is not influenced by the natural ASA pattern. In fact, it's going in the opposite direction (Supplementary Figure S19).

### 4.5 Time vs. type of surgery

The Case Total average time is inversely related to SSR. The HRA, with the highest SSR, takes the most time, and UKA takes the least amount of time. However, the variance in time is not nearly as significant as it is for SSR when it comes to different types of surgeries. There are significant similarities between the two knee surgeries and two hip surgeries, respectively. The only outlier is the average APT which is significantly higher in TKA (Supplementary Figure S20). The APT average for hip procedures is significantly lower compared to knee operations but has minimal to no impact on the Case Total average. Procedure time and APT in Room may have the most significant influence on the Case Total average.

## 5 Level-3: staff vs. patient vs. time-metrics analysis

At the highest level, we are analyzing four variables at once to identify more complex patterns in data and correlations that are invisible or not credible enough at Level-1 or Level-2. We are analyzing two-time metrics and one staff/Patient metric with SSR. Different four-variable combinations can help us identify a wealth of insights and trends via a comprehensive descriptive analysis.

### 5.1 Surgeon-SSR vs. procedure and SPT averages

We are comparing surgeons' (with their respective SSR) numbers for their procedure time average (highly relevant to surgeons) and SPT (less relevant to surgeons). In this scenario, one extreme would be the surgeon with a high SST and low procedure average, and the other would be a high procedure time and low SST. Surgeon PB is an example of the positive extreme, but they have also benefited from low average SPT. Surgeon GD is an example of the other extreme who, despite having low SPT, had high procedure times and low SSR (Supplementary Figure S21). Surgeons (with their respective SSRs) were plotted for the following (X and Y axis) variables:

• SFT and Average of AFT (Clustering—SFT 3–6 and AFT 9–16)
• AFT and APT (Clustering—AFT 20–40 and APT 9–16)
• APTinRoom and SPT (Clustering—APTinRoom 10–16 and SPT 13–16)

However, no discernable pattern was observed, apart from clustering in certain intersections of the above-stated variables. Recall that it was the same for the circular nurses when they were plotted against turnover, APTinRoom, and SFT.

### 5.2 Surgeon-SSR vs. BMI and age

The most successful surgeon (based on procedure time and SSR) has operated on patients with the lowest average BMI and age of all surgeons. However, this is not the case for the other extreme. Age seems to have a far more significant impact on the average time a surgeon takes to complete a procedure than BMI. However, the limitations of this analysis should be considered with reference to the sample size (more surgeons to compare). A greater sample with multiple data points concentrated within a specific age range (like below 60 or above 75) can cast a shadow on the strength of this correlation (Supplementary Figures S22, S23).

## 6 Discussion

Descriptive analytics (DA) helps us decipher raw data from the past and identify patterns and trends to generate useful insights that may be applied to future decision-making. By identifying relationships between different metrics (variables), it helps us differentiate the most crucial metrics from relatively non-important ones that may not have a significant enough impact on trends and SSR. Comprehensive DA and the identification of the most important metrics can become the foundation for more advanced Diagnostic Analytics, which focuses on the reasons and rationales behind certain trends, i.e., the “why” behind what happened (14).

Understanding how different metrics/variables interact with each other and how they impact SSR and metrics that directly influence SSR (Time Metrics: Complete Case Time and Procedure Time) can lead to efficient operating room (and even emergency room) decision-making. Identifying the most high-impact metrics and learning how small changes to them can lead to significant improvement in the SSR can help clinical institutions develop low-cost, low-effort strategies to achieve more on-time surgery completions. An example is changing the teaching day, which requires minimal effort and no cost and can have an enormous impact on the SSR.

Our comprehensive descriptive analytics of the data collected from the 4-joint Arthroplasty surgeries at our institution revealed the following insights. Note that these insights are selected from dozens of individual analyses performed on the collected data points.

• If we analyze the three sets of metrics (staff, patient, and time) based on how strongly they influence/impact the SSR, staff metrics take the lead. Patient metrics had the most minimal impact on the SSR.
• Time in Room for the first case of the day influences the SSR of the rest of the cases. Hence it is very important to start the day on time.
• Even though it may seem logical that the most experienced professionals (especially surgeons), with the highest number of surgeries on their record, would complete more procedures on time than their less experienced peers, the analysis revealed that it was not the case. The experience of medical professionals had almost no impact on the SSR, our analytics says it could be their patient selection or their surgical time or both.
• The SSR jumped as high as 45% from the least successful month (14% SSR) to the most successful one (59% SSR).
• The variance among SSR on different days of the week is less significant than months, but it's still significant, i.e., 19%. The highest SSR for a day reached 46%, while the lowest was around 27%.
• On average, male patients required five more minutes per procedure compared to female patients.
• The age and ASA classification of the patients had a significant impact on anesthesia metrics but not on the overall procedure duration.

It's important to understand that many of the above observations are limited by the spread of data which may have influenced the accuracy of some resulting patterns. Most of the outliers are in the extremes. For age, the bulk of the data points is concentrated between the ages of 47 and 76. For BMI, most data points/patients fall between 22.1 and 27.1. As for ASA, most patients are classified as Class II or III, with only a fraction in Class I or IV. Sex is the only variable that's safe from this uneven spread.

DA also helps us identify unique and useful patterns and trends that emerge from the data by combining and comparing a couple of metrics together and studying their relationship (17). However, the effectiveness of DA goes down as dimensionality (i.e., the number of variables/columns of data) increases. It becomes difficult to identify patterns and trends to generate useful insights. Another limitation of DA is the number of variables it can simultaneously handle (18). Some insights can only be generated when more variables are being analyzed at once, and that is where more comprehensive analytical techniques (predictive and prescriptive analytics) and machine learning comes into play (19).

Predictive and prescriptive analytics can take dozens of variables/parameters/dimensions into account and simultaneously analyze them to identify more complex and insightful patterns. Machine learning algorithms are significantly more powerful and can handle thousands of parameters and variables at once. This sophistication allows them to determine patterns and generate insights that DA is unable to generate, though it doesn't undermine its usefulness.

Another reason more sophisticated analytical techniques and machine learning algorithms are prioritized over DA is the depth of analysis. Since it can only handle a few variables and dimensions at once, many of the insights generated are naturally shallow and may simply lead to ineffective or resource-intensive actions taken to achieve a desired outcome. In the worst cases, the conclusions may be wrong and lead to potentially damaging decisions. Using these insights to infer cause and effect without exploring the deeper relationships of these variables to others may lead to wrong conclusions (20).

This also limits the portability of decision-making frameworks based on DA. The decisions and conclusions of a DA may not apply to a different healthcare setting and cannot be generalized for a broader range of scenarios (21). They are usually only valid for the data at hand, and decisions made using the DA can only effectively apply to the source of the data (in this case, the 4-joint surgical OR).

As DA is rooted in the past, it neither informs us about the future nor helps us predict how changes in the current variables will influence the future. This is the domain of predictive analytics, which gives us a glimpse of the future and helps us positively influence it by making relevant changes.

This limitation is tied to the DA's inherent limitation of identifying what happened (patterns) but not why and how it happened. Since it doesn't identify the cause that leads to the apparent effect (pattern/trend), its effectiveness is limited when it comes to decision-making. In contrast, ML algorithms like Decision Tree and Linear Regression that also incorporate DA's strength (explainability) shed a more comprehensive light on the past, and the insights they reveal can be applied to future decision-making (achieving the desired output).

## 7 Conclusion

The insights generated in our study endorse an important benefit of descriptive analysis, i.e., identifying high-impact metrics. Various analyses can help with the identification of the highest-impact metrics and prevent researchers from assigning more weight to variables/metrics that may seem more impactful than they are due to cognitive bias (like staff experience). In conclusion, the insights can help influence the re-allocation of resources in daily clinical practice to offset inefficiencies in arthroplasty surgeries.

## Data Availability

The original contributions presented in the study are included in the article/**Supplementary Material**, further inquiries can be directed to the corresponding author.
