# Supplementary material for: Factors influencing delays and overtime during surgery: a descriptive analytics for high volume arthroplasty procedures
Source: Front Surg. 2024 Jan 4;10:1242287. doi: 10.3389/fsurg.2023.1242287 (PMC10797887; doi:10.3389/fsurg.2023.1242287)
Supplement: Supplementary file 1 [file Datasheet1.docx]

Supplementary Material

Factors Influencing Delays and Overtime During Surgery: A Descriptive Analytics for High Volume Arthroplasty Procedures

Farid Al Zoubi^*^, Paul Beaule, Pascal Fallavollita

* Correspondence: Farid Al Zoubi, [falzo100@uottawa.ca](mailto:falzo100@uottawa.ca)

# Supplementary Figures


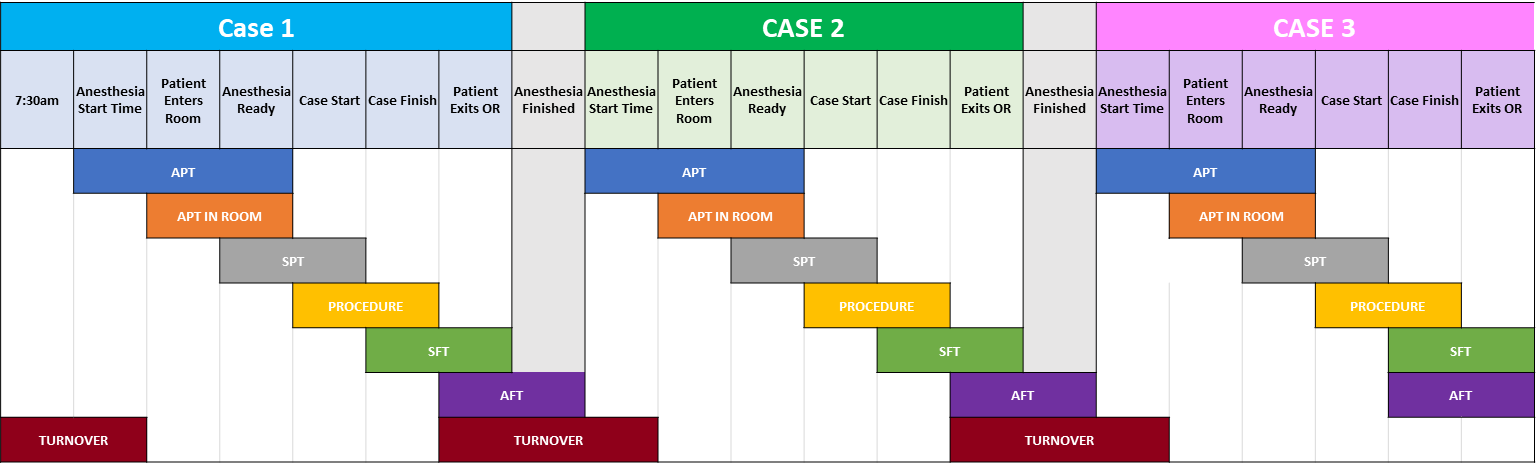


Figure 1: Surgical workflow steps [9]


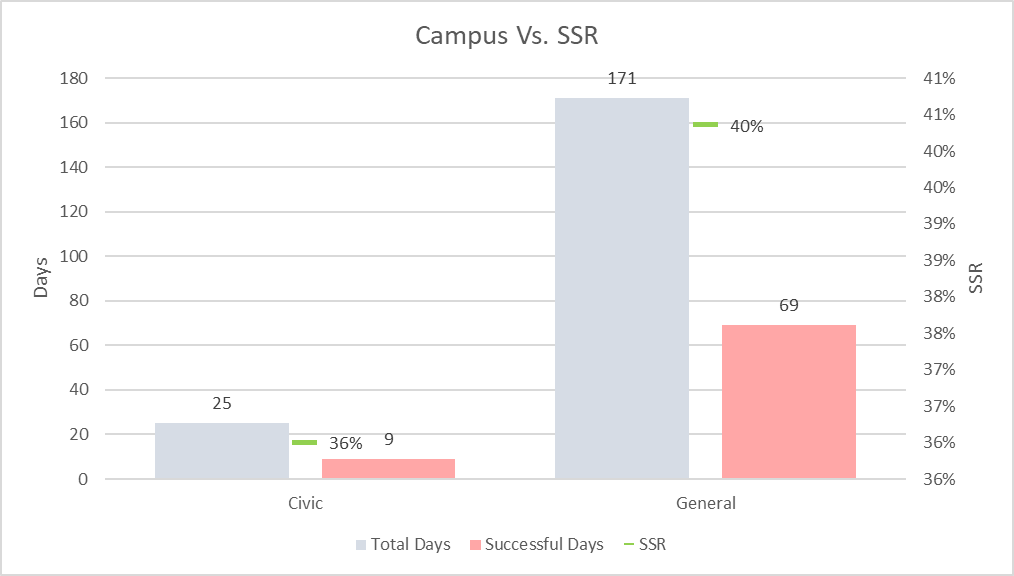


Figure 2: Average SSR for both campuses is 39%.


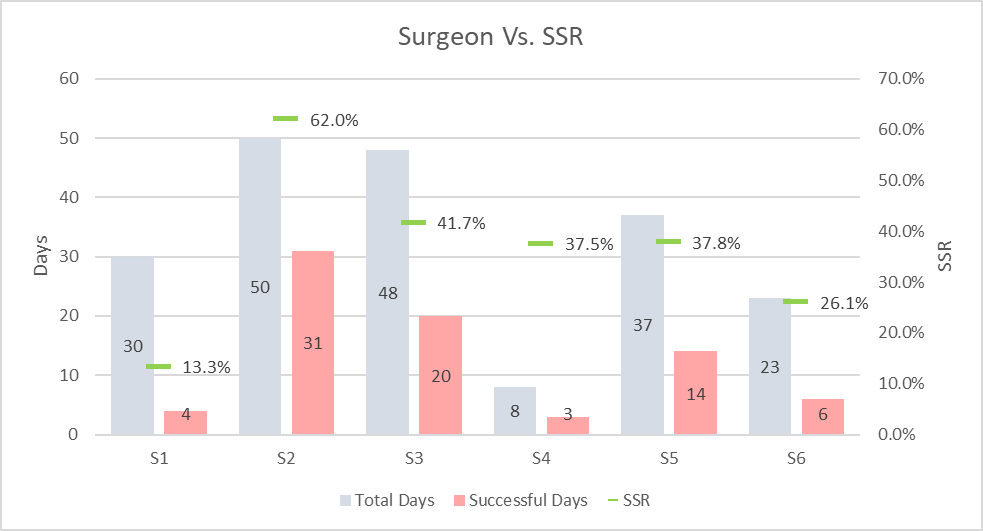


Figure 3: Surgeon’s experience did not influence SSR.


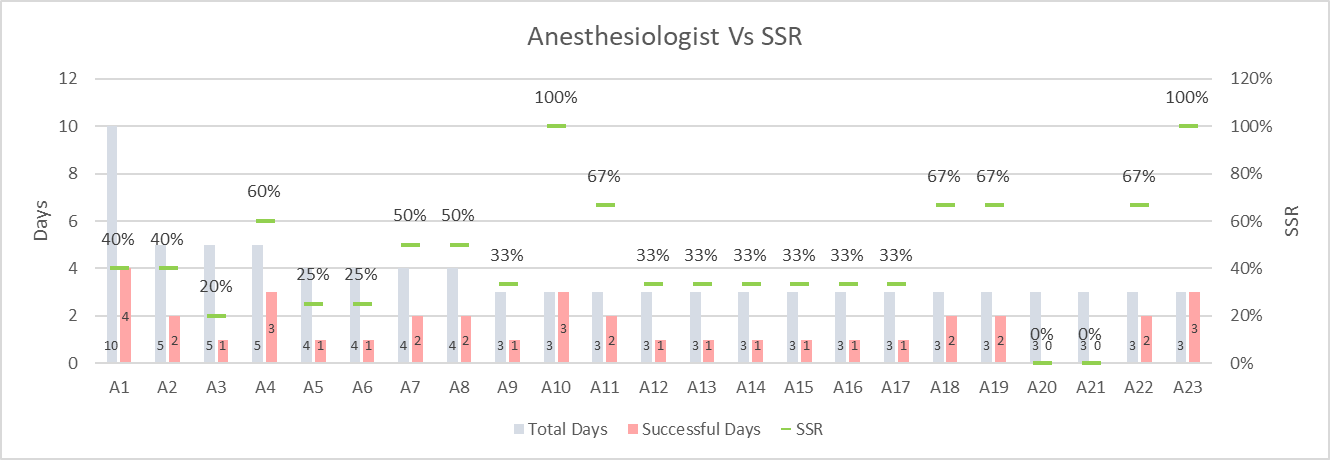


Figure 4: Anesthesiologist’s experience did not influence SSR.


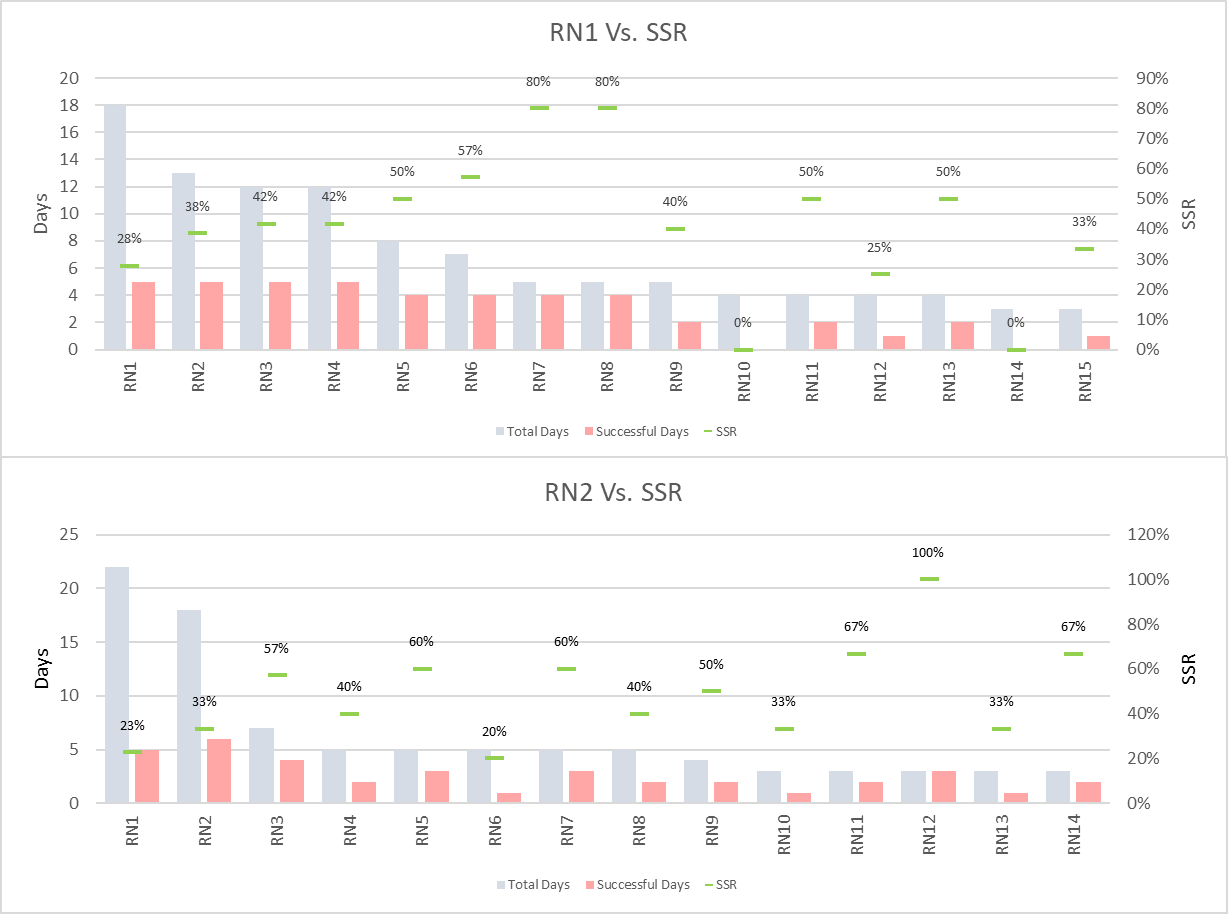


Figure 5: Circulating nurse’s experience did not influence SSR.


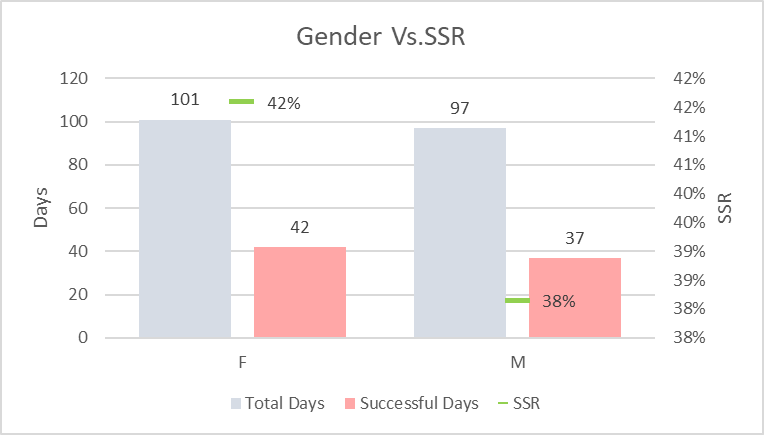


Figure 6: four percent difference in SSR between male and female patients.


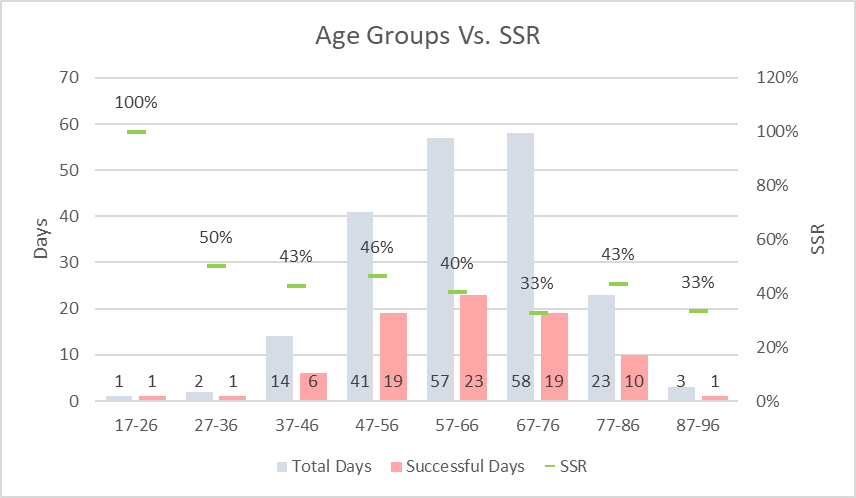


Figure 7: Differences in SSR between patient age groups.


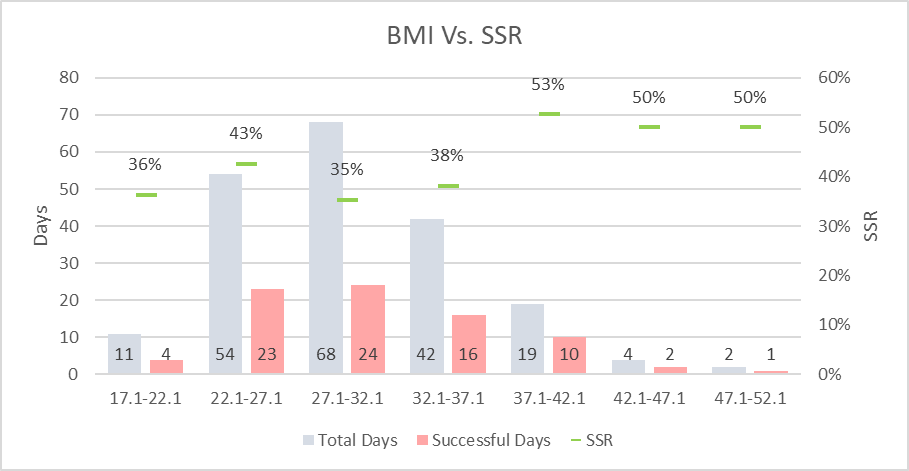


Figure 8: BMI and SSR did not follow same pattern.


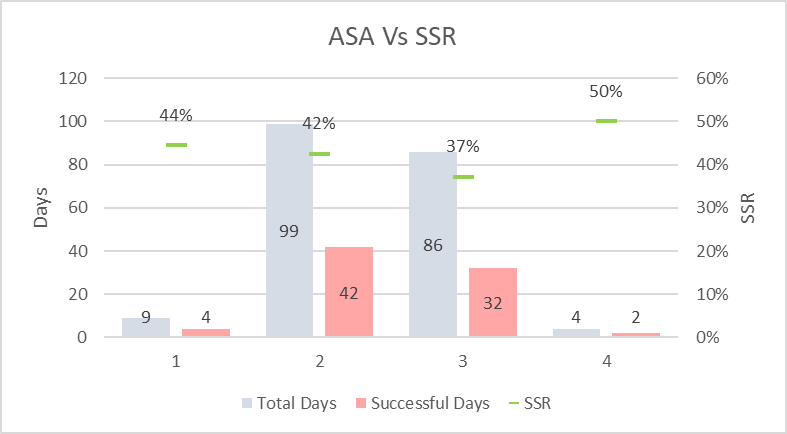


Figure 9: SSR is higher for a safer ASA class and lower for a riskier class.


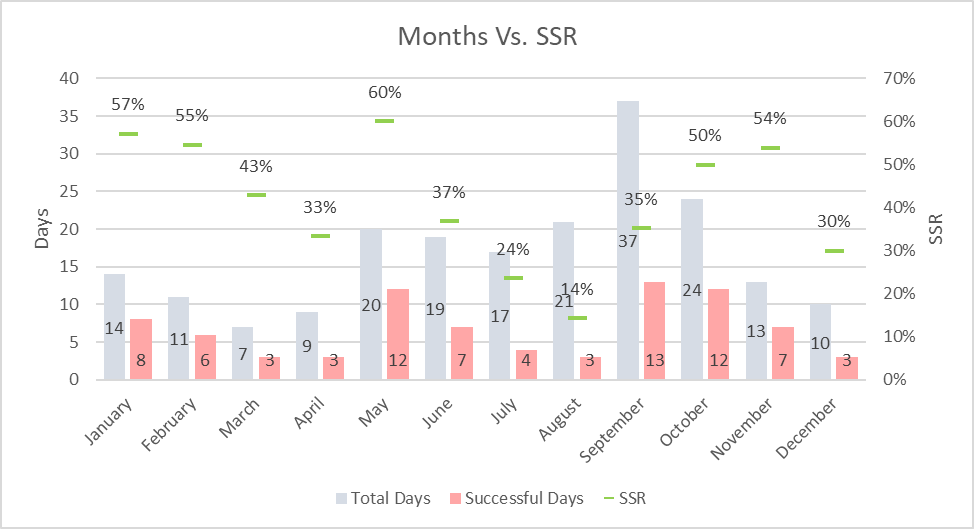


Figure 10: The month of May has the highest SSR opposite to the month of August.


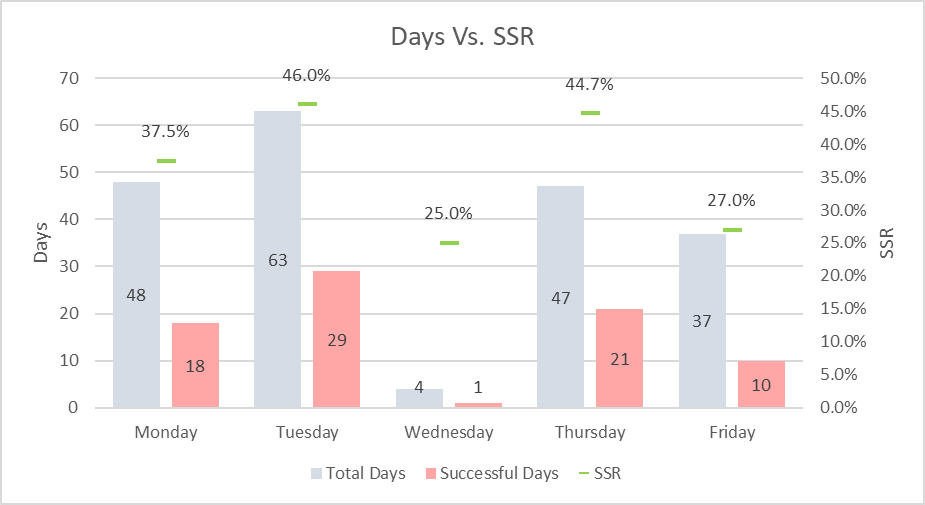


Figure 11: Days after and before weekends have the worst SSR.


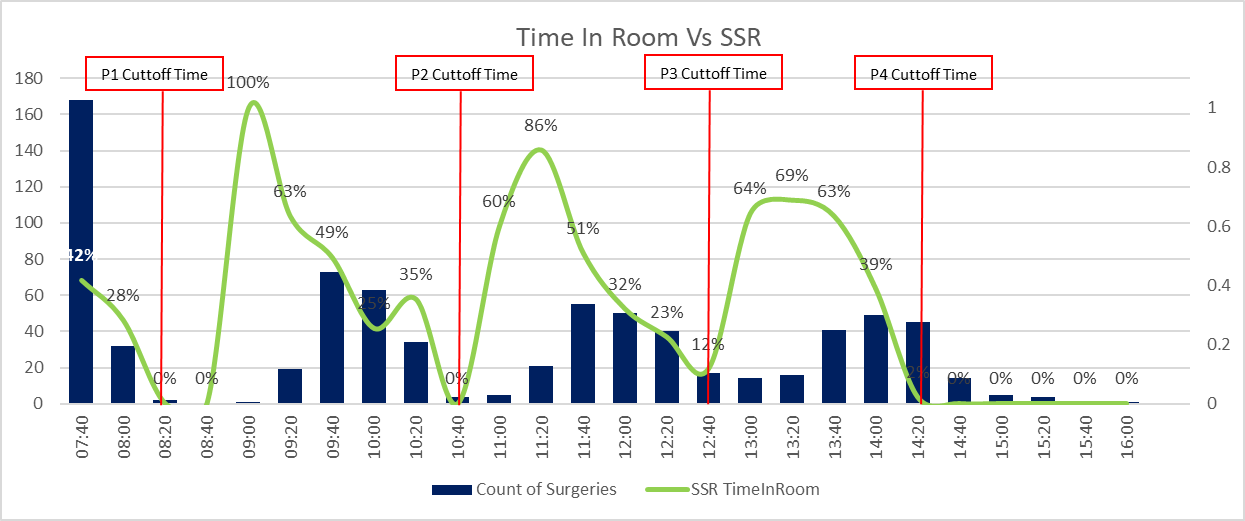


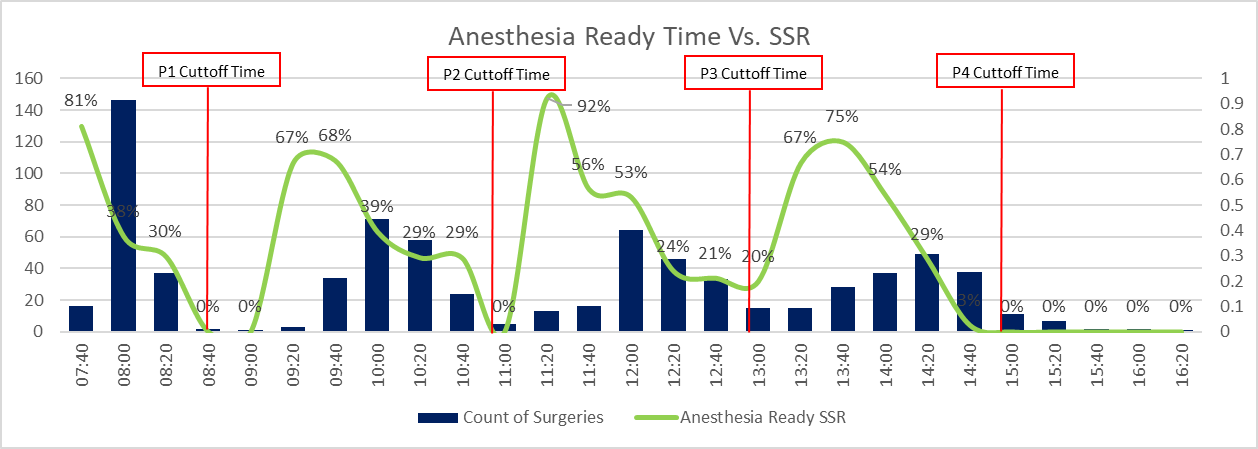


Figure 12: The SSR is higher for surgeries in which the patient was in the room and anesthetized close to the scheduled time.


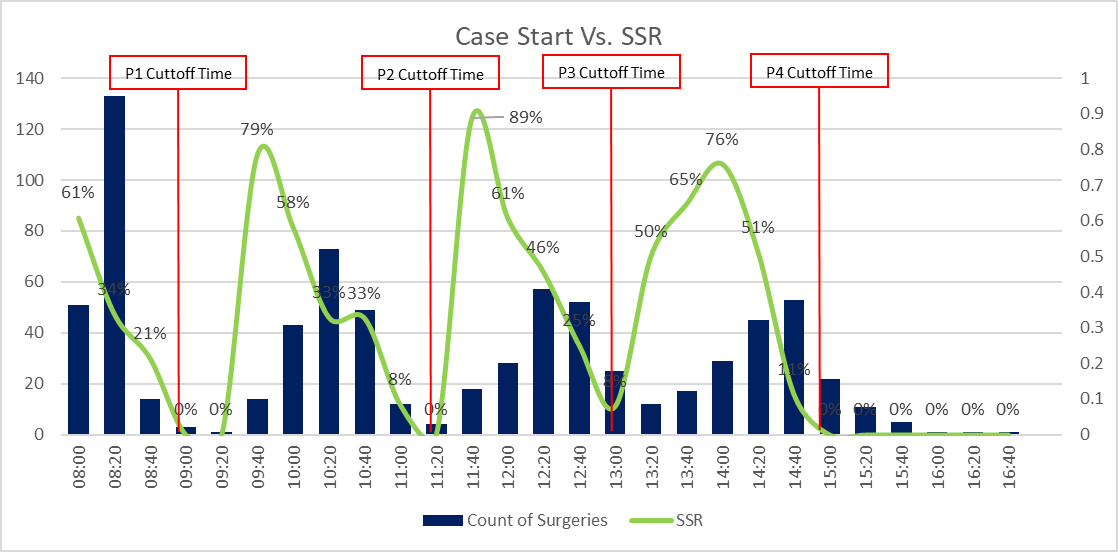


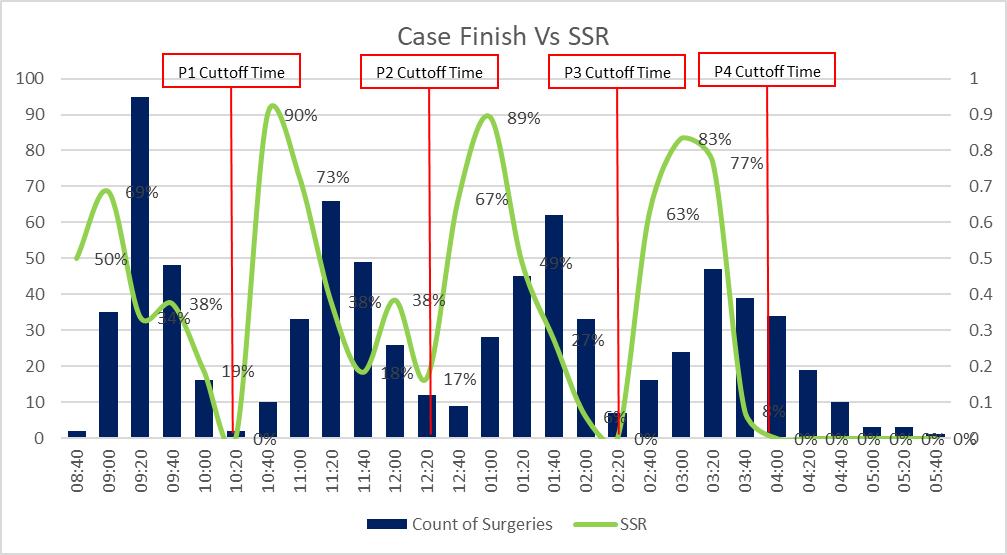


Figure 13: Procedures that started at the allotted time slots had a much higher SSR rate


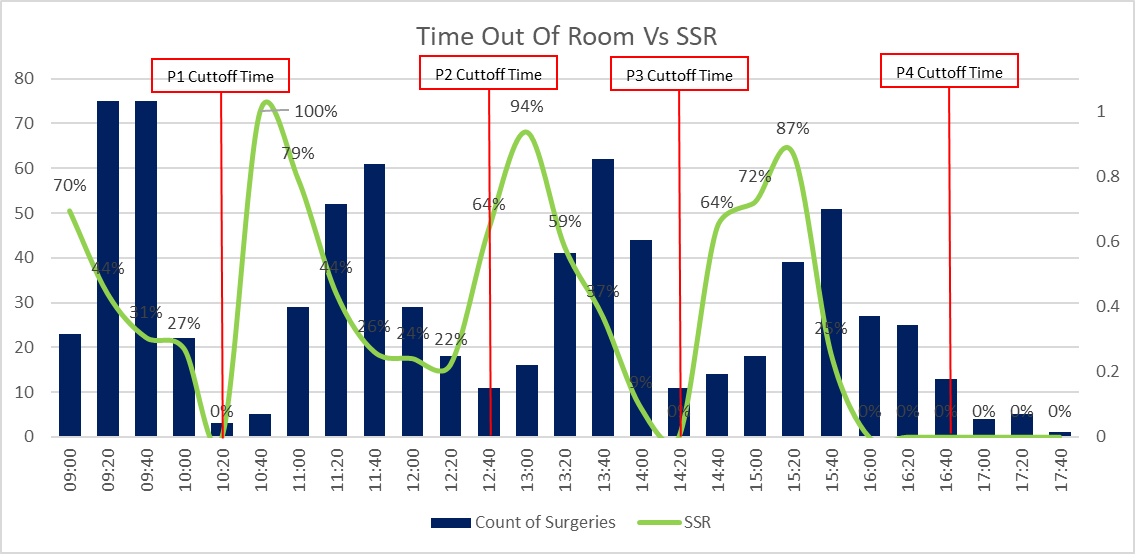


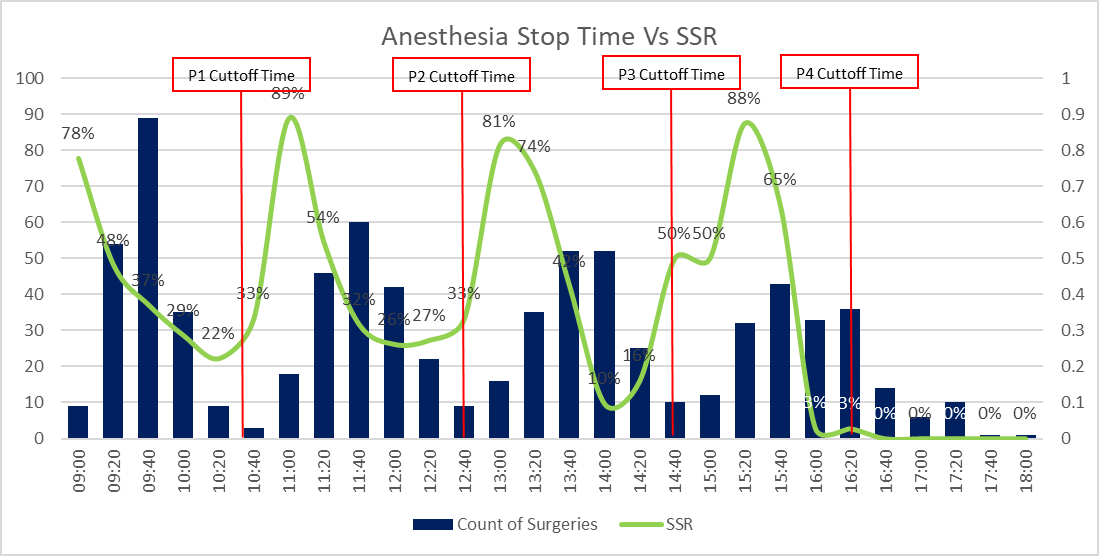


Figure 14: The earlier the (out of room/anesthesia stop) times the higher SSR.


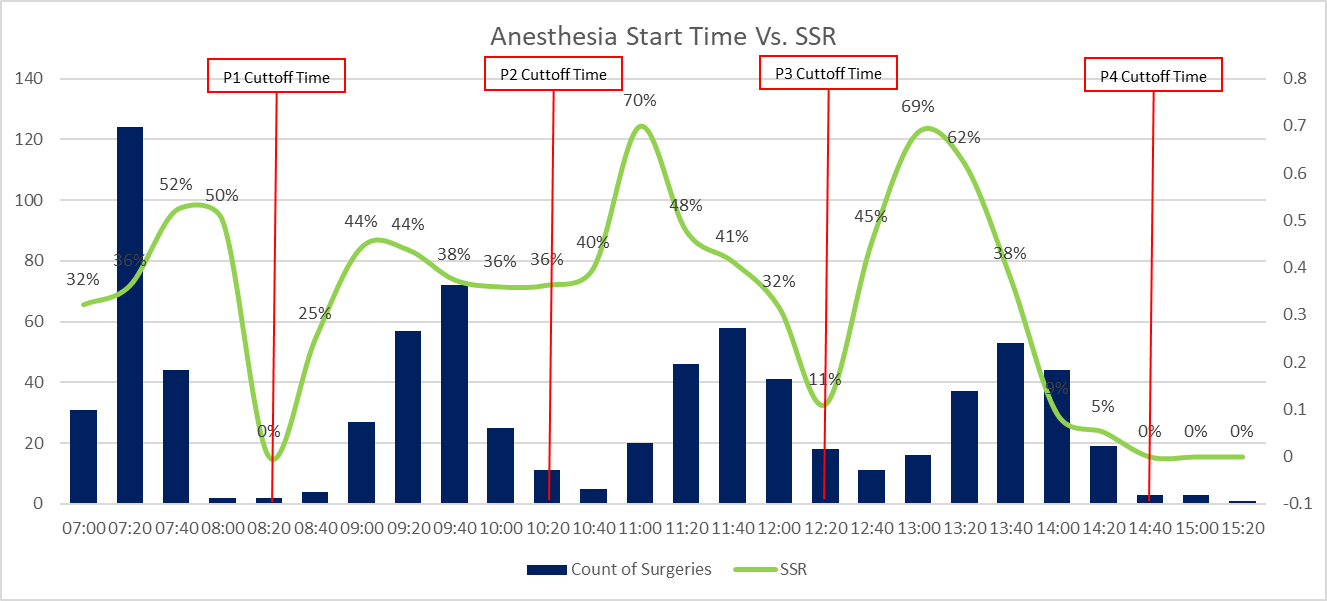


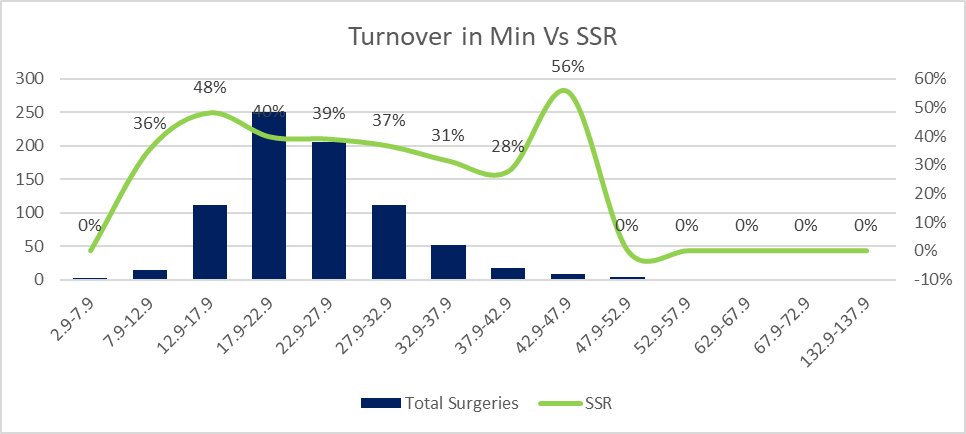


Figure 15: Different patterns are observed for turnover (in minutes) and anesthesia start time.


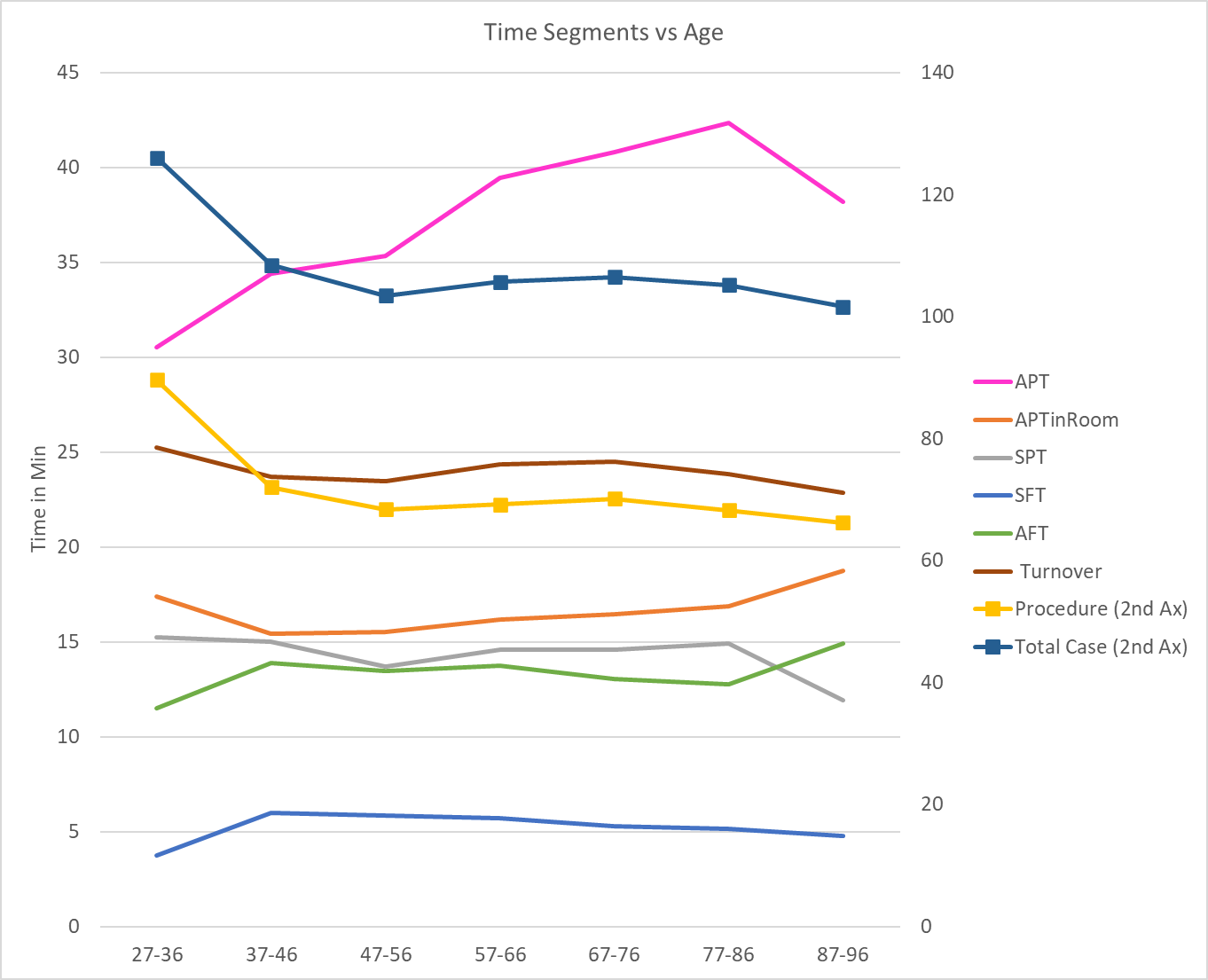


Figure 16: Anesthesia preparation and in-room time go up with age.


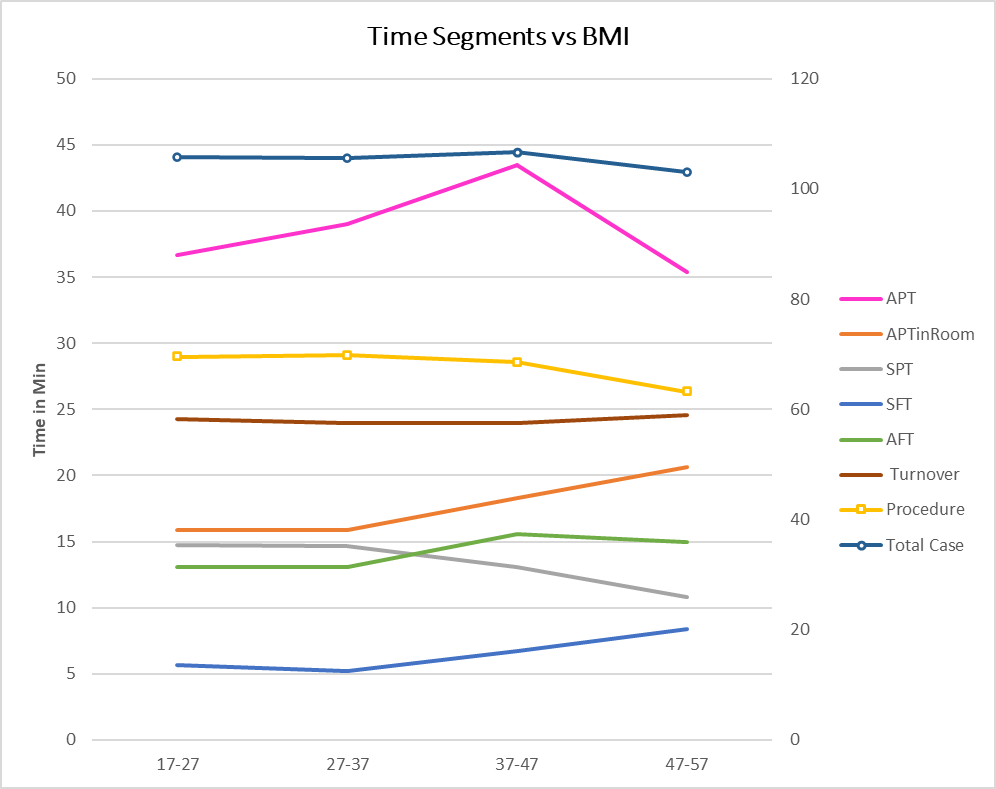


Figure 17: Relationship between BMI and time metrics for surgery.


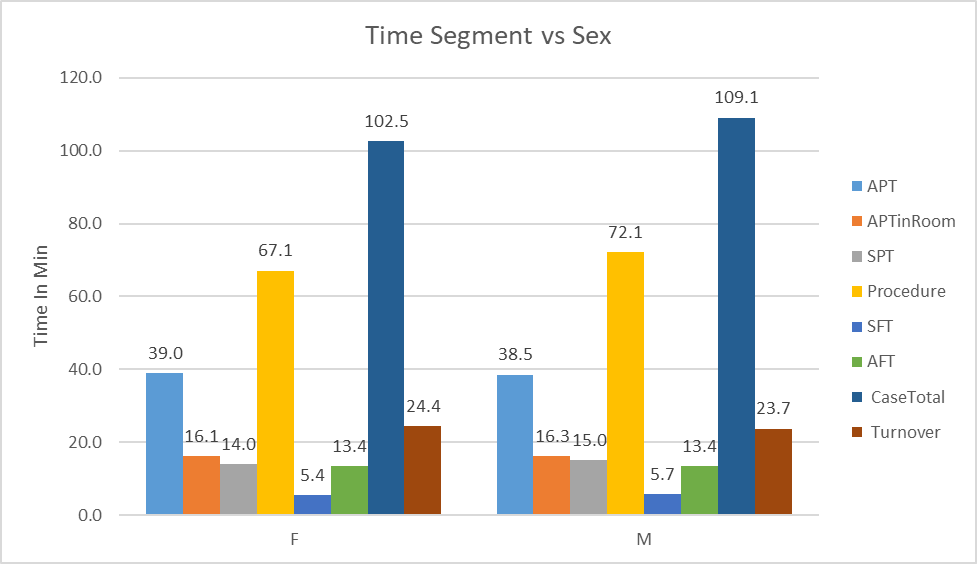


Figure 18: Male patient surgeries are on average 5 minutes longer than those for female patients.


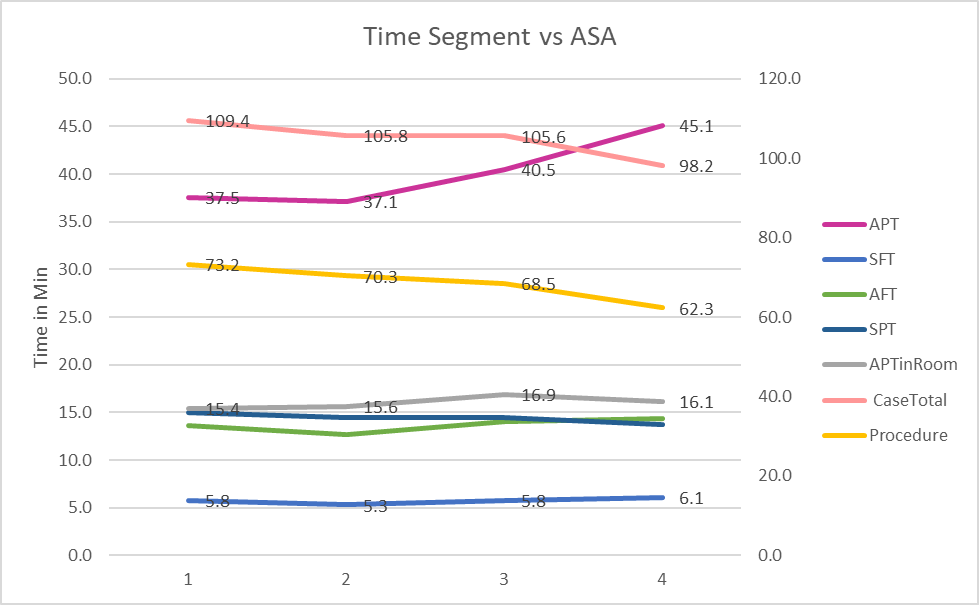


Figure 19: Only APT is directly positively correlated to ASA.


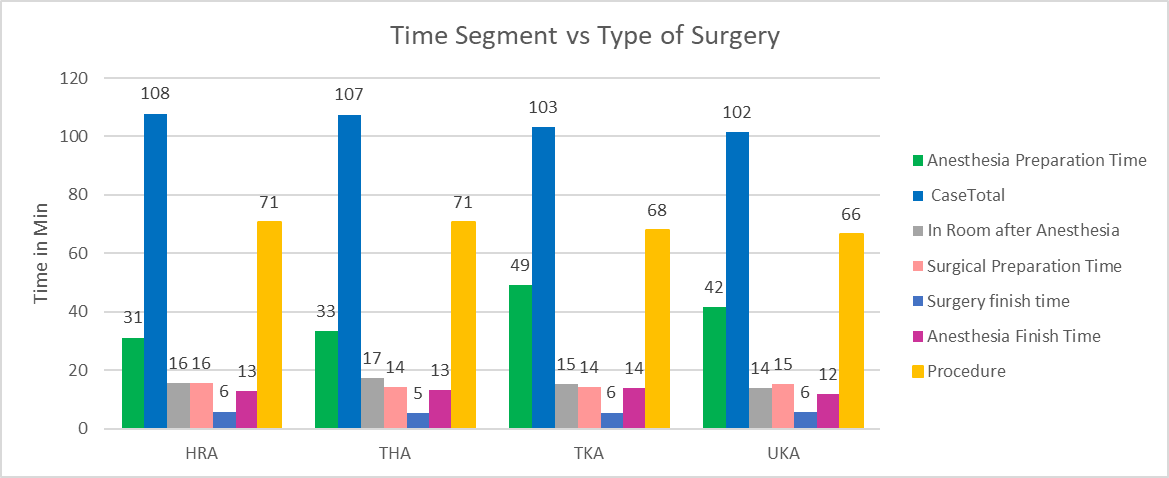


Figure 20: Variance in time is not significant as it is for SSR for different types of surgeries.


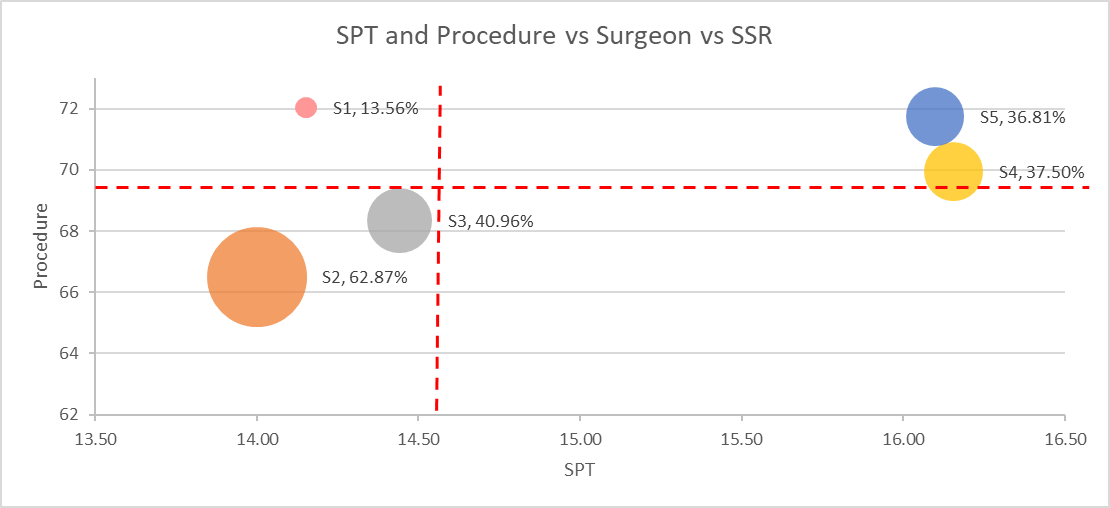


**Figure 21:** Surgeons, in general, with high SSR have lower SPT and procedure time. The size of the sphere represents the SSR of the surgeon.


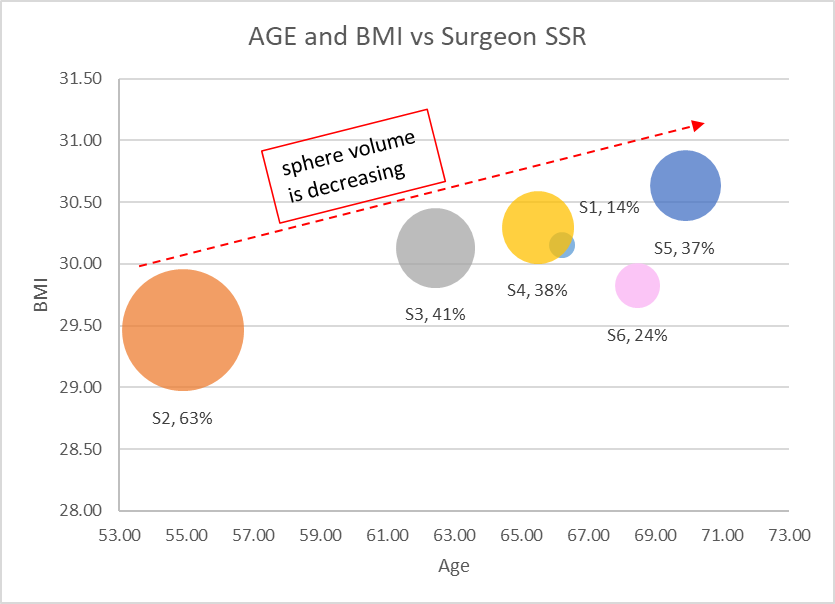


**Figure 22:** Age is more important on surgeon SSR than BMI.


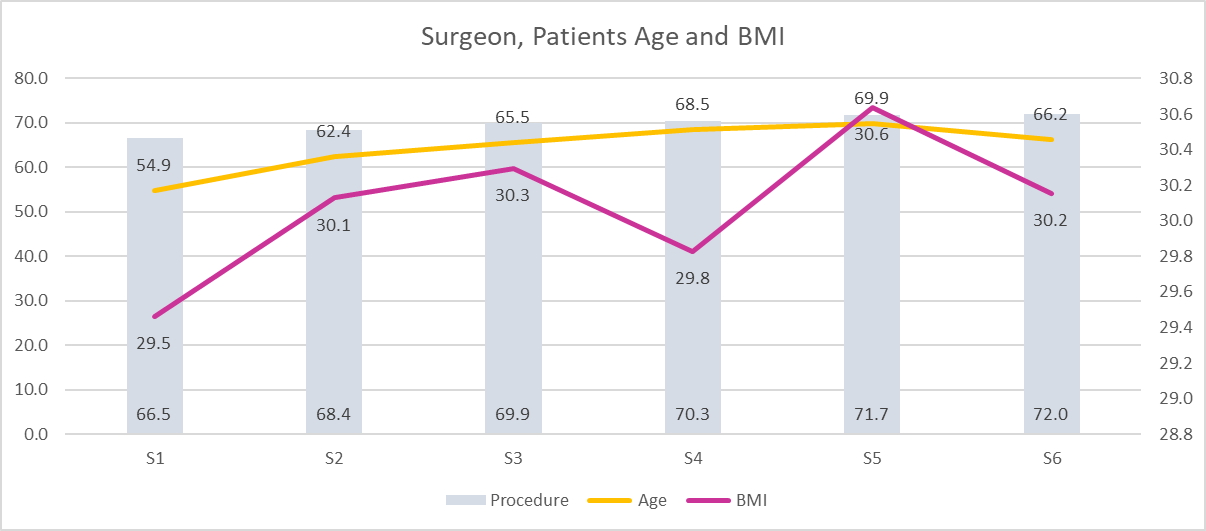


**Figure 23:** Surgeons with higher SSR have operated on patients with lower BMI and age.

# Supplementary Tables

| Time Metrics | Staff (Team) Metrics | Patient Metrics | Safety metrics |
| --- | --- | --- | --- |
| Anesthesia Preparation time | Surgeon | Campus | 90-day Readmissions |
| Anesthesia Preparation time in Room | Anesthesiologist | Type of Surgery | Reason for Readmission |
| Anesthesia Start time | Circulator Nurse 1 | Type of Anesthesia | Length of Stay |
| Time in Room | Circulator Nurse 2 | Sex |  |
| Anesthesia Ready time |  | Age |  |
| Anesthesia Stop Time |  | BMI |  |
| Anesthesia Finish Time |  | ASA |  |
| Surgical Preparation Time |  |  |  |
| Case Start |  |  |  |
| Case Finish |  |  |  |
| Surgery finish time |  |  |  |
| Turnover |  |  |  |
| Surgery (Procedure) Time |  |  |  |
| Time Out of Room |  |  |  |
| Case no |  |  |  |
| Date |  |  |  |

**Table 1**: Metrics and their categories

| **Metric** | **Pearson Correlation With SSR** | **Spearman Correlation With SSR** |
| --- | --- | --- |
| **Age** | -0.088 | -0.092 |
| **ASA** | -0.038 | -0.046 |
| **Turnover** | -0.079 | -0.069 |
| **APT** | -0.181 | -0.195 |
| **AFT** | -0.067 | -0.085 |
| **APTinRoom** | -0.094 | -0.109 |
| **BMI** | 0.018 | 0.004 |
| **SFT** | -0.107 | -0.124 |
| **SPT** | -0.106 | -0.109 |
| **Procedure** | -0.150 | -0.197 |
| **Sex** | -0.031 | -0.031 |

**Table 2:** Correlation of each numerical metric to the SSR
